# Failure of GLP-1 Agonist Therapy to Improve Weight in a 3-Year-Old Patient With Tumor-Related Obesity

**DOI:** 10.1155/crpe/1707315

**Published:** 2025-11-20

**Authors:** Rebecca Petlansky, Evan Graber

**Affiliations:** ^1^Department of Pediatrics, Nemours Children's Health, Wilmington, Delaware, USA; ^2^Division of Endocrinology, Nemours Children's Health, Wilmington, Delaware, USA

**Keywords:** abnormal weight gain, hypothalamic obesity, obesity treatment, tumors of CNS

## Abstract

A 19-month-old female patient presented due to rapid weight gain starting at age 5 months. Due to continued abnormal weight gain after 1 year of age, an MRI of the brain was performed, revealing a 5.5 × 2.6 × 2.3 cm mass centered within the medulla oblongata with extension to the C3 vertebral body. Biopsy confirmed a diagnosis of ganglioglioma. A trial of topiramate was attempted, but there was no improvement in weight gain after 2 months. The patient started on 0.3 mg liraglutide daily, and the dose was titrated up to 3 mg daily over 3 months. The patient did not experience abdominal pain, nausea, vomiting, or diarrhea. Weight continued to increase at a drastic rate, and increased appetite persisted. Liraglutide was discontinued. Liraglutide may be safe for use in children as young as age 3 years. However, its effect on weight loss in cases of hypothalamic obesity may be limited. The medulla oblongata contains GLP-1 receptors and is involved in the suppression of food intake. In the presented case, lack of response to treatment was likely due to medullary damage from the tumor. Further research about the etiology of neurogenic causes of obesity is needed so targeted therapies may be developed.

**Summary**

• GLP-1 agonist use is likely not a reasonable option for use in weight gain due to CNS tumors.
• GLP-1 agonist use for nontumor-related obesity may be safe in children as young as 3 years.
• The brainstem is involved in energy balance, and consideration should be given to renaming hypothalamic obesity to neurogenic obesity.

## 1. Introduction

Hypothalamic obesity is seen in conditions where energy balance signals in the hypothalamus are disrupted due to various etiologies including damage from tumor infiltration, trauma, or cranial irradiation to name a few [1]. Neurons involved in energy balance are also located in the solitary nucleus (NTS) and area postrema (AP) in the medulla oblongata of the brainstem, but damage to these structures is rarely discussed when considering abnormal weight gain [2]. Glucagon-like peptide-1 (GLP-1) is an incretin hormone secreted by enteroendocrine cells in the small intestine that enhances insulin secretion, inhibits glucagon release, slows gastric emptying, and plays a key role in appetite suppression and decreasing food intake by stimulating GLP-1 receptors in the hypothalamus and brainstem [3]. GLP-1 receptor agonists are approved for use in the United States for treatment of Type 2 diabetes mellitus and obesity in children and adolescents aged 10 years and older. This case demonstrates the use of GLP-1 agonist therapy in a 3-year-old patient with tumor-related weight gain due to a ganglioglioma located in her medulla oblongata.

## 2. Case Presentation

The patient was a previously healthy full-term female who presented to her primary care provider at 5 months of age due to rapid weight gain and breath-holding spells. She was first evaluated by endocrinology at 19 months of age for ravenous appetite, food-seeking behaviors, and rapid weight gain despite diet and lifestyle changes including working with a registered dietician, applying locks to food-containing cabinets, and limiting snacks, portion sizes, and screen time.

### 2.1. Diagnostic Assessment

**Figure 1:**
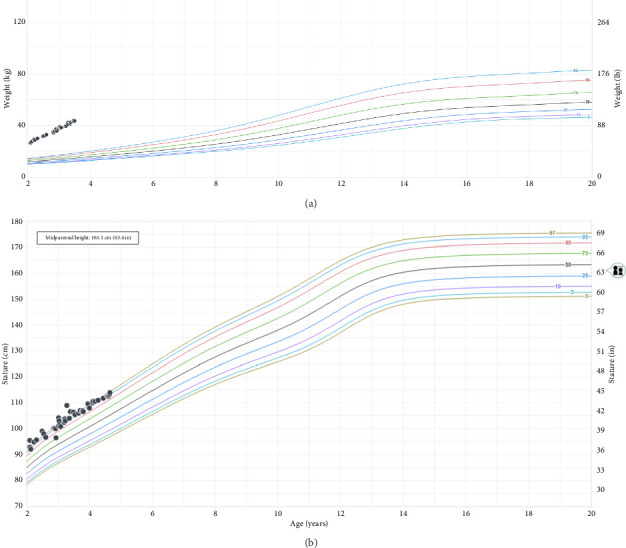
(a) Patient weight-for-age chart adapted from the Centers for Disease Control and Prevention (CDC) for girls aged 2–20 years. (b) Patient stature-for-age adapted from CDC for girls aged 2–20 years.

Examination was remarkable for severe obesity without dysmorphisms; hemihypertrophy, striae, and brachydactyly were absent. Her growth chart showed that weight increased rapidly from the 90th percentile at age 5 months to > 99th percentile by age 18 months, and linear growth was normal. This pattern continued throughout her evaluation (Figure 1).

Due to normal growth velocity, normal development, and failure of lifestyle interventions to improve weight, a genetic cause of obesity was considered. Evaluation for monogenic causes of obesity demonstrated a variant of uncertain significance in the single-minded 1 (*SIM1*) gene. However, as the patient's unaffected father was found to have the same variant, this change was thought unlikely to be the cause of her symptoms. Methylation testing for Prader–Willi/Angelman syndromes, genetic sequencing for syndromic macrocephaly/overgrowth syndromes, chromosomal microarray, and whole exome sequencing were nondiagnostic. Pituitary hormone testing was performed as shown in Table 1.

**Figure 2:**
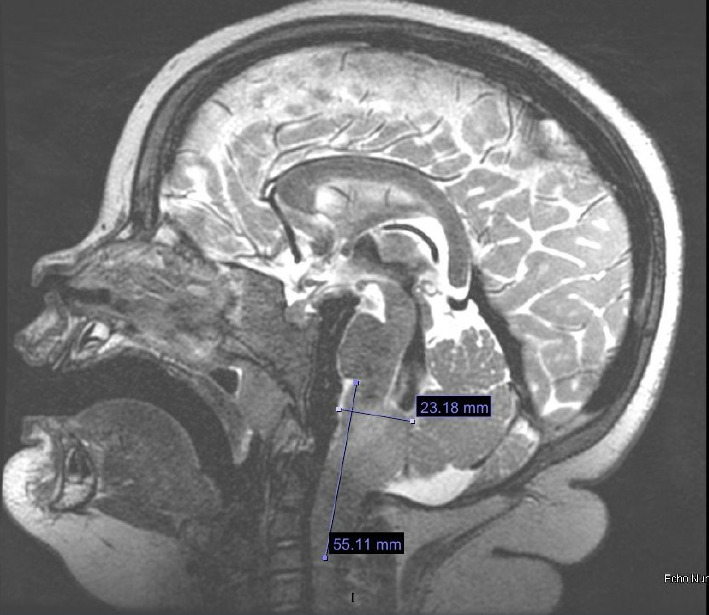
T2-weighted magnetic resonance imaging demonstrating the patient's medullary mass. Subsequent biopsy was consistent with ganglioglioma.

At age 2 years 11 months, in the absence of other explainable etiologies of her ongoing weight gain, an MRI of the brain and spine was obtained that revealed a 5.5 × 2.6 × 2.3 cm mass centered within the medulla oblongata with extension to the level of the C3 vertebral body with an exophytic component along the left aspect of the medulla. Biopsy was consistent with ganglioglioma (Figure 2).

### 2.2. Treatment

The patient started on a trial of topiramate. The dose was titrated up to 75 mg twice daily, but there was no change in the rate of weight gain. Topiramate was discontinued after about 2 months of use.

Due to continued weight gain and complications from her weight (sleep apnea, hypertension, and leg pain), liraglutide therapy was discussed and initiated at age 3 years. The family was counseled about the off-label use of liraglutide at this age as well as potential side effects, and they agreed with proceeding. There was no concern for familial medullary thyroid cancer or history of pancreatitis in the patient. She started on a dose of 0.3 mg subcutaneously daily, and the dose was titrated up to a maximum dose of 3 mg daily over a 3-month period.

### 2.3. Outcome and Follow-Up

**Figure 3:**
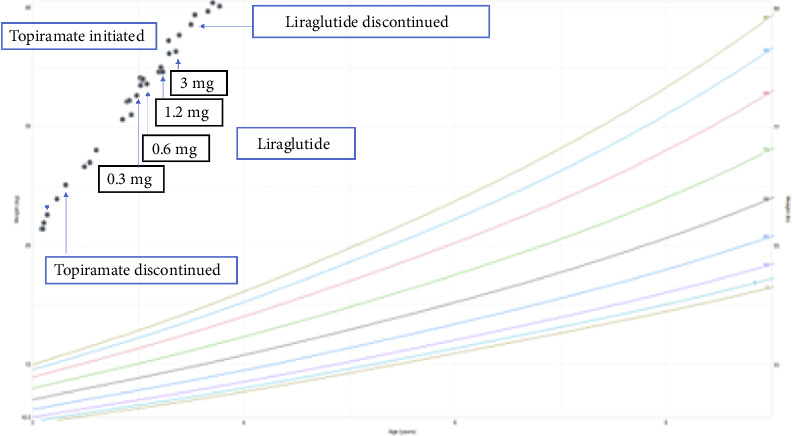
Patient weight-for-age chart adapted from CDC for girls aged 2–20 years with labels indicating start of topiramate, discontinuation of topiramate, start of liraglutide, and discontinuation of liraglutide.

The patient did not experience any abdominal pain, nausea, vomiting, or diarrhea. There was an initial parental perception of a decrease in the patient's appetite, but this did not last longer than 1 month of therapy. Weight continued to increase at a drastic rate, and increased appetite persisted. Liraglutide was discontinued after about 6 months (Figure 3). Tirzepatide has been initiated. Treatment and monitoring are ongoing.

## 3. Discussion

Hypothalamic obesity is a well-known phenomenon that is associated with suprasellar tumors (up to 60% of patients with craniopharyngioma will develop hypothalamic obesity) or treatment for brain tumors that lie close to the hypothalamus [1]. Disruption of satiety signals results in increased appetite and rapid weight gain that are difficult to treat. Many medications, including stimulants that increase energy consumption, diazoxide, antidiabetic agents, and octreotide, have been suggested as treatments for hypothalamic obesity, but none to date have shown efficacy in significantly reducing weight and/or are associated with significant side effects [4–7]. Studies are also often limited by small sample size. In contrast to obesity caused by hypothalamic damage, the role of the brainstem in energy balance is less often discussed. However, the NTS and AP in the medulla oblongata are also involved in food intake suppression and contain GLP-1 receptors. GLP-1 is an incretin secreted by enteroendocrine cells in the small intestine that enhances insulin secretion, inhibits the release of glucagon, and slows gastric emptying. GLP-1 also acts on satiety centers of the brain, resulting in appetite suppression and decreased food intake [3].

Disruption of neural pathways in the brainstem is associated with monogenic obesity syndromes that appear similar to hypothalamic weight gain [8, 9] (Table 2). This is likely, at least in part, due to disruption of GLP-1 signaling. Targeted treatments for some monogenic obesity conditions have been developed. Congenital leptin deficiency and leptin receptor deficiency may present soon after birth due to mutations of leptin (*LEP)* or its receptor (*LEPR)*. Leptin acts in the arcuate nucleus of the hypothalamus to stimulate production of alpha-melanocyte-stimulating hormone (α-MSH), which then acts downstream as a satiety signal through activation of the melanocortin 4 receptor (MC4R). Treatment of leptin deficiency may include subcutaneous injection of a leptin analog (metreleptin), though this is not effective in leptin receptor mutations. Setmelanotide, an MC4R agonist, may be used to treat pathogenic mutations in *LEPR, POMC* (which encodes the prohormone precursor to MSH), or *PCSK1* [10]. Without targeted treatment, monogenic causes of obesity are treated similarly to other causes of pediatric obesity.

Guidelines for the treatment of children and adolescents with obesity have been outlined by the American Academy of Pediatrics [11]. Treatment is longitudinal and involves evaluation and treatment of comorbidities, identification of social determinants of health, and motivational interviewing surrounding nutrition and physical activity. Recommendations for pharmacotherapy are also included. Orlistat, an intestinal lipase inhibitor that blocks fat absorption through inhibition of pancreatic and gastric lipase, is FDA-approved for long-term treatment of obesity in children 12 years and older. Phentermine is FDA-approved for short-course therapy (3 months) for adolescents 16 years or older. Phentermine inhibits central norepinephrine uptake inhibitor as well as serotonin and dopamine reuptake and reduces appetite. Topiramate is a carbonic anhydrase inhibitor and suppresses appetite centrally through largely unknown mechanisms. Topiramate is currently FDA-approved for the treatment of headache in pediatrics. Topiramate is used in combination with phentermine in adults. Lisdexamfetamine is a stimulant approved for children 6 years and older with ADHD that is occasionally used off-label for the treatment of obesity. Given its modest and inconsistent effectiveness, metformin may be considered if other comorbidities, such as Type 2 diabetes mellitus or polycystic ovarian syndrome, are present. Most recently, GLP-1 receptor agonists have been found to be helpful in improving weight in those with exogenous weight gain. A randomized controlled trial published in 2020 found liraglutide more effective than placebo in weight loss at 1 year among patients 12 years and older with obesity who did not respond to lifestyle treatment, and liraglutide was subsequently approved for use for weight loss in children and adolescents [11, 12]. However, studies of GLP-1 agonist use in weight gain due to hypothalamic or brainstem damage are lacking.

This case is the youngest reported GLP-1 agonist use and the first attempted use for weight gain related specifically to a brainstem tumor. Since 2020, GLP-1 receptor agonists have become a valuable resource for managing weight gain in patients 10 years and older. In the case reported, weight gain was so dramatic that GLP-1 receptor agonist therapy was attempted in a child younger than the age at which this class of drugs has been thoroughly studied. The medication was not effective in slowing the patient's weight gain, likely due to damage of medullary satiety centers that contain GLP-1 receptors. Despite the lack of efficacy, the patient tolerated the medication and did not have any adverse side effects, including abdominal pain, nausea, vomiting, or diarrhea. This was true even at the maximum dose of 3 mg.

Fox et al. performed a randomized controlled trial examining the use of liraglutide in children aged 6 to less than 12 years with obesity [13]. Liraglutide, in doses up to 3 mg daily, was found to result in a significant reduction in BMI in children treated with lifestyle interventions and liraglutide compared to placebo. However, gastrointestinal side effects were common, occurring in 80% of the intervention group compared to 54% in the control group. There have not been studies examining GLP-1 use in children younger than 6. It is not clear why our patient was able to tolerate GLP-1 agonist therapy at such a high dose despite side effects being so common. Although abdominal discomfort is often attributed to the decreased gastric emptying caused by GLP-1 agonists, one must consider whether CNS stimulation may also be involved as the NTS plays a role in the development of nausea and vomiting. It may be that in patients with CNS damage such as ours, there could be less propensity to developing gastrointestinal side effects due to the same damage that reduces the weight loss effect.

The patient was found to have a paternally inherited single nucleotide variant of uncertain significance in the *SIM1* gene (c.1720A > G) discovered on a genetic obesity panel. This gene is expressed during the development of the hypothalamic–pituitary axis in the paraventricular nucleus, the anterior periventricular nucleus, and the supraoptic nucleus [14]. Michaud et al. demonstrated that mice with heterozygous mutations in the *SIM1* gene display hyperphagia and develop early-onset obesity [15]. Similarly, loss-of-function mutations in humans have been associated with obesity with or without a Prader–Willi-like syndrome [16]. Although this gene is involved in the same pathways that ultimately lead to this patient's obesity, the mutation is a variant of uncertain significance and was also present in the unaffected father. It is unclear whether this mutation may have also contributed to this patient's weight gain.

In conclusion, GLP-1 agonist use may not be a reasonable option for use in weight gain due to CNS tumors. This may be due to permanent damage to neural networks involved in energy balance and satiety signals, whether they occur in the area of the hypothalamus or the brainstem. Further research about the etiology of hypothalamic obesity is needed so targeted therapies may be developed. In nontumor-related obesity, GLP-1 agonist use may be safe in children younger than 6 years, but additional studies are needed to determine the effectiveness and safety profile. It is important to recall that the brainstem is involved in energy balance, and consideration should be given to renaming hypothalamic obesity to neurogenic obesity to account for the multiple brain centers involved in pathologic weight gain.

## Figures and Tables

**Table 1:** Patient laboratory data.

Date of study	TSH	Free T4	AM cortisol	ACTH	Insulin	IGF-1	IGF-BP3	Prolactin	Leptin
4/20/2022			7.5 μg/dL (206.9 nmol/L)						
7/27/2022	1.79 mIU/L (0.028 nmol/L)	1.3 ng/dL (16.7 pmol/L)			13.3 μIU/mL (79.8 pmol/L)				
11/18/2022	1.41 mIU/L (0.022 nmol/L)	1.1 ng/dL (14.2 pmol/L)	14 μg/dL (386.3 nmol/L)	27 pg/mL (5.9 pmol/L)		146 ng/mL (19.08 nmol/L)	4.3 mg/L (149.6 nmol/L)		
4/19/2023	2.9 mIU/L (0.045 nmol/L)	1.4 ng/dL (18 pmol/L)		47 pg/mL (10.4 pmol/L)		149 ng/mL (19.47 nmol/L)		24.6 ng/mL (24.6 μg/L)	18.2 ng/mL (1.14 nmol/L)

*Note:* ACTH = adrenocorticotropic hormone, IGF1 = insulin-like growth factor 1, IGFBP3 = insulin-like growth factor-binding protein 3.

Abbreviation: TSH = thyroid-stimulating hormone.

**Table 2:** Genetic causes of human obesity with encoded protein function and phenotype of affected individuals.

Gene	Pattern	Function	Phenotype
LEP	Monogenic	Activates POMC neurons in the arcuate nucleus of the hypothalamus to produce alpha-MSH.	Severe obesity starting days after birth, hypogonadotropic hypogonadism, central hypothyroidism, and immunologic abnormalities
LEPR	Monogenic	Leptin receptor at which leptin activates POMC neurons.	Severe obesity starting days after birth, hypogonadotropic hypogonadism, central hypothyroidism, growth hormone deficiency, and immunologic abnormalities
POMC	Monogenic	Precursor protein to melanocyte-stimulating hormone (MSH) and adrenocorticotropic hormone (ACTH)	Severe obesity starting months after birth, central adrenal insufficiency, mild hypothyroidism, red hair, and pale skin
PCSK1	Monogenic	Expressed in neuroendocrine tissues where it is involved in processing of hormone and neuropeptide precursors including POMC, proinsulin, and proglucagon	Severe obesity starting in childhood, central adrenal insufficiency, hypogonadotropic hypogonadism, growth hormone deficiency, central hypothyroidism, central diabetes insipidus, postprandial hypoglycemia, and malabsorptive diarrhea
MC4R	Oligogenic	Located in the paraventricular nucleus of the hypothalamus, produces satiety signal when activated by alpha-MSH	Variable severity of obesity, estimated to be involved in 2%–3% of obese children and adults
SIM1	Monogenic	Transcriptional factor involved in the development of the hypothalamic–pituitary axis in the paraventricular nucleus, the anterior periventricular nucleus, and the supraoptic nucleus	Severe obesity starting in childhood, variable neurobehavioral differences, and Prader–Willi-like symptoms

*Note:* LEP = leptin, LEPR = leptin receptor, POMC = proopiomelanocortin, PCSK1 = proprotein convertase subtilisin/kexin type 1, MC4R = melanocortin 4 receptor, SIM 1 = single-minded 1.

## Data Availability

Original data generated and analyzed during this study are included in this published article.
